# Remission trajectories and cognitive function in hospitalized youth with depressive episodes

**DOI:** 10.3389/fpsyt.2025.1673240

**Published:** 2025-10-01

**Authors:** Jie Yang, Nanxi Li, Xuequan Zhu, Ziheng Zhang, Sijia Chang, Yinbing Zhang, Haochu Gong, Ming Dong, Haibo Wang, Longjun Cai, Xiaoyi Wang, Gang Wang

**Affiliations:** ^1^ National Clinical Research Center for Mental Disorders & National Center for Mental Disorders, Beijing Anding Hospital, Capital Medical University, Beijing, China; ^2^ Beijing Wispirit Technology Co., Ltd, Beijing, China; ^3^ Institute of Advanced Clinical Medicine, Peking University, Beijing, China; ^4^ Clinical Research Institute, Key Laboratory of Epidemiology of Major Diseases, Peking University, Ministry of Education, Beijing, China

**Keywords:** adolescent, depressive episodes, cognitive function, trajectories, hospitalized

## Abstract

**Background:**

Depressive episodes in adolescents and young adults are a significant global health concern, marked by high prevalence, cognitive impairments, and elevated suicide risk. Despite their clinical importance, remission trajectories and cognitive function in hospitalized youth remain understudied, particularly in transdiagnostic contexts.

**Methods:**

This retrospective cohort study analyzed electronic health records from 792 hospitalized patients (aged 13–22) with depressive episodes, using the Hamilton Depression Rating Scale (HDRS-17) and the Primary Cognitive Ability Test (PCAT III) to assess symptom trajectories and cognitive function. Gaussian Mixture Models identified distinct remission patterns, while linear mixed-effects models evaluated associations between depression severity, cognitive domains, and clinical factors.

**Results:**

Three trajectory groups emerged: Severe-Rapid Remission (7.7%), Moderate-Rapid Remission (15.3%), and Moderate-Slow Remission (77.0%). Working memory was related to depression severity, and anxiety symptoms were associated with cognitive performance. Additionally, patients diagnosed with bipolar depression showed reduced performance in both language comprehension and working memory at baseline. Intensive treatments (e.g., electroconvulsive therapy) showed efficacy but highlighted variability in response.

**Conclusion:**

The findings suggest that tailored interventions addressing baseline severity, anxiety, and cognitive support may be beneficial in hospitalized youth, with possible diagnostic relevance for bipolar depression.

## 1 Introduction

Depressive episodes, a typical type of mood episode in affective disorders, are characterized by persistent low mood, loss of interest, and accompanying cognitive and physiological symptoms. Depressive episodes are particularly significant during adolescence and young adulthood (1). Depression in children and adolescents may influence depression symptoms and compromised psychosocial functioning in young adulthood (2). According to the Global Burden of Disease (GBD) study, the average prevalence of mental disorders among individuals aged 5–24 is 11.63% (3). Notably, the prevalence of mood disorders increases sharply from early to late adolescence (4). Additionally, the point prevalence of elevated depressive symptoms among adolescents has risen 13% in 10 years (5). In China, the total prevalence of mental disorders among children and adolescents is 17.5% (6). Depressive episodes in this demographic not only have a high incidence but also exhibit unique disease characteristics, such as fluctuating symptoms, comorbid anxiety, and substance abuse (7). These episodes are often accompanied by severe social and functional impairments, which presented as declined academic performance (8) and worsened interpersonal relationships (9). More critically, this population faces a significantly elevated risk of suicide, with studies indicating that suicide is the second leading cause of death among adolescents and young adults (10), and depressive episodes are a major risk factor for suicidal behavior (11).

Mood disorders that begin in childhood may present as complex arrays of symptoms during adolescence, making it difficult to classify them under a single diagnostic category (12). Research indicates that 50% of patients initially diagnosed with depression may have bipolar disorder (BD), with the proportion potentially higher in this age group (13). Previous studies have largely been confined to single-disease diagnoses or specific populations, such as non-suicidal self-injury (14), potentially overlooking transdiagnostic patterns (15), particularly in cognitive functioning. Cognitive function is especially crucial for adolescents and young adults, as their still-developing prefrontal cortex and hippocampus provide heightened plasticity for attention, memory, and executive function (16). However, depressive episodes can significantly disrupt this developmental process. Existing studies have shown that cognitive impairments are widespread among depressive patients (17), including reduced attention, impaired working memory, and executive dysfunction. These deficits hinder academic performance, problem-solving, and decision-making, worsening social functioning and self-efficacy (17). Thus, focusing on the state of depressive episodes rather than specific disease diagnoses in cognitive function research for this age group may more comprehensively capture the commonalities of cognitive impairments and provide more universally applicable guidance for clinical practice (18).

Acute-phase treatment for depressive episodes is crucial for improving patient prognosis. Most young people with depression can be managed as outpatients. However, hospitalization is required when outpatient care cannot control self-harm or suicidal risk, particularly when psychotic symptoms are present (19), or when progressively intensive outpatient treatment remains ineffective. Hospitalization allows closer monitoring than outpatient care, capturing dynamic and nuanced trajectories of depressive episodes (20), and providing more frequent and detailed tracking of symptoms and treatment response. Despite the clinical importance of investigating short-term illness trajectories in this hospitalized population, research specifically examining adolescents and young adults remains notably limited. Child and adolescent depressive symptoms are related to adult mood disorders (21). Analyzing real-world data to explore these outcome trajectories and their influencing factors is essential for advancing our understanding of depressive episodes. By clarifying these trajectories, clinicians can optimize treatment plans, improve short-term patient outcomes, and ultimately enhance long-term functional recovery (22).

This study aims to observe the trajectory of symptom changes in hospitalized adolescents and young adults with depressive episodes. Additionally, we will analyze the factors influencing cognitive function and investigate the relationship between depressive symptoms and cognitive function. The innovation of this study lies in its focus on the trajectories of depressive episode patients in the stages of adolescence and early adulthood, addressing a gap in the existing research on this specific population. By elucidating the influencing factors of and the relationship between cognitive function and depressive symptoms, it contributes to a deeper understanding of the manifestations of cognitive impairments in depressive episodes.

## 2 Methods

### 2.1 Participants and follow-up

This retrospective cohort study used electronic health records based on the Hospital Information System of Beijing Anding Hospital from June 2022 to May 2024. Beijing Anding Hospital (a tertiary Grade-A psychiatric hospital, the highest level in China’s hospital classification system) serves both local Beijing residents and patients throughout northern China, with approximately 50% of patients coming from outside Beijing.

**Figure 1:**
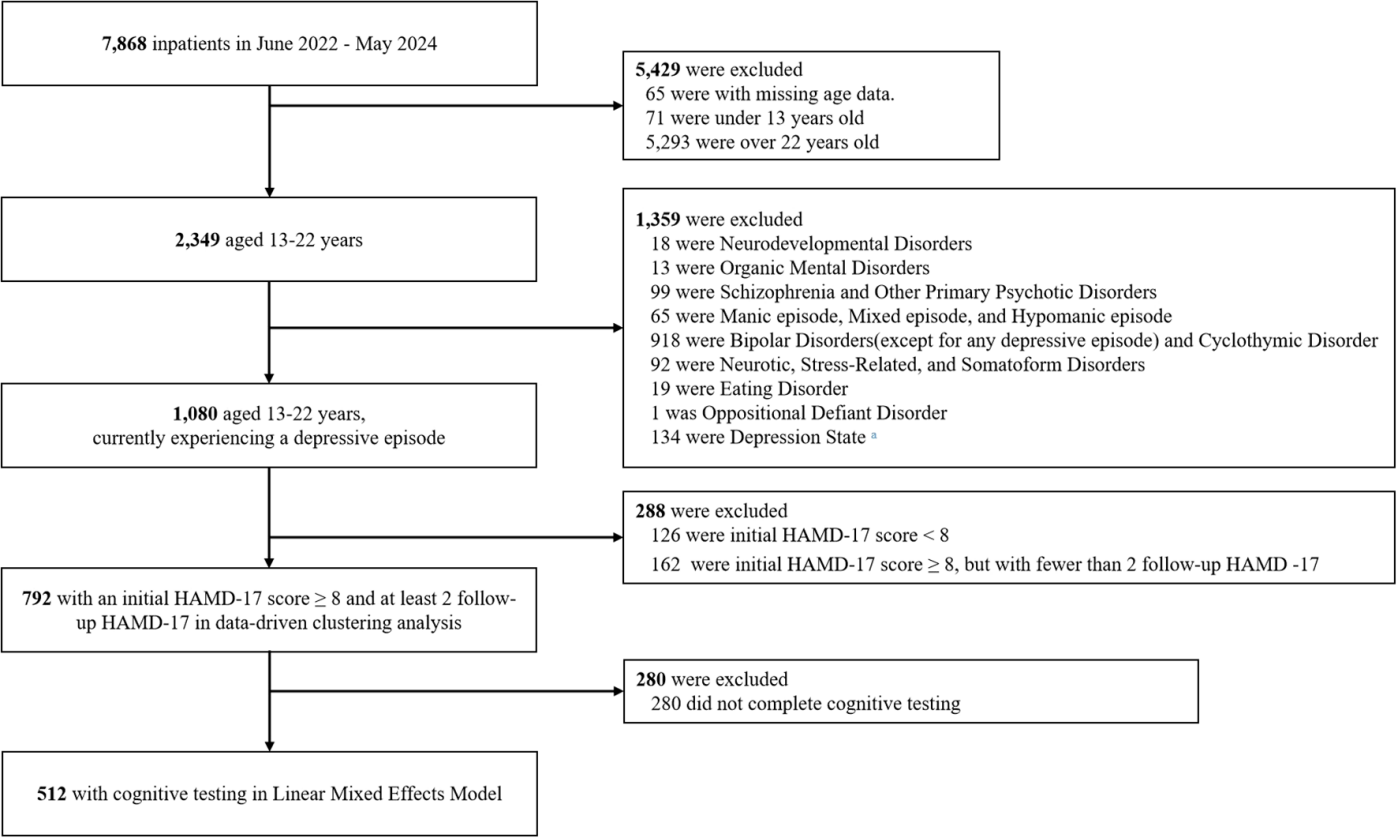
Study flowchart. ^a^ Patients were initially diagnosed with depressive state and were discharged within 3 days without definitive diagnostic classification.

The International Statistical Classification of Diseases and Related Health Problems, Tenth Revision (ICD-10) (23), classification lacks specific diagnostic categories for adolescent mood disorders. Although mood disorders with onset in childhood or adolescence are classified under F30-F39, many young patients present with chronic, non-episodic irritability superimposed on depressive mood rather than classical manic/hypomanic symptoms. As such, these cases often fail to meet standard criteria for typica mood disorder and are diagnosed as Unspecified behavioral and emotional disorders with onset usually occurring in childhood and adolescence (UBED-CA; ICD F 98.9), with initial clinical presentations labeled as depressive state. The diagnostic gap was partly addressed by the introduction of disruptive mood dysregulation disorder (23) in the Diagnostic and Statistical Manual of Mental Disorders, Fifth Edition (24), which established a distinct entity within depressive disorders. To enhance both clinical validity and population representativeness in capturing this understudied patient group, UBED-CA was included as one of the diagnostic criteria in this study. Based on this diagnostic framework, the main eligibility criteria were as follows: (1) Diagnosed with depressive episode, including Bipolar Disorder (BD) with depressive episodes (F31.3 - F31.5), Depressive episodes (F32.0 - F32.3), Recurrent depressive disorder (F33.0 - F33.3), Dysthymia (F34.1); (2) Initial presentation of depressive state who were later diagnosed with UBED-CA (F98.9); (3) Aged 13–22 years old; (4) 17-item Hamilton Depression Rating Scale (HDRS-17) scores of at least 8 on three or more occasions during hospitalization. **Figure 1** shows the study flowchart. The study protocol received approval from the Ethics Committee of Beijing Anding Hospital, Capital Medical University (2024-Research-156). The individual data were anonymous, exempting the study from requiring informed consent.

**Figure 2:**
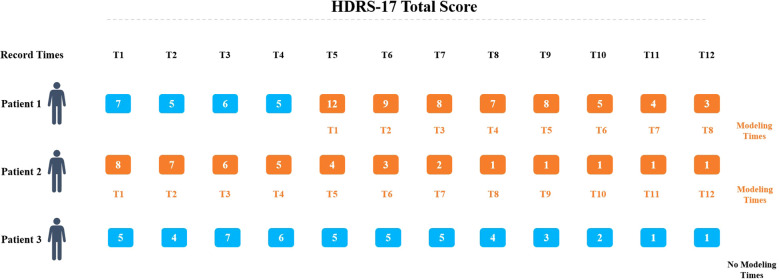
Model baseline definition. The baseline was defined as the first HRSD-17 score ≥8 during hospitalization, with ≥2 subsequent assessments required. The nearest cognitive assessment within ±7 days of baseline was selected.

In the study, all the patients underwent an initial HDRS-17 assessment on admission day. The first cognitive assessment was administered on the second hospital day, adjusting for off-hours admissions (e.g., evenings, weekends, or Chinese public holidays). In these cases, cognitive testing was deferred to the next regular weekday. To ensure assessment validity, no patient received Modified Electroconvulsive Therapy (MECT) before their initial cognitive evaluation. We established a baseline using the first HDRS-17 score of 8 or higher recorded during hospitalization. To be included, participants needed at least two additional HDRS-17 assessments after baseline as shown in **Figure 2**. For cognitive function, we selected the assessment closest to the baseline date from those conducted within a 14-day period (7 days before or after baseline). We excluded patients who had fewer than three HDRS-17 scores during their hospital stay, whose maximum HDRS-17 score never reached 8, or who lacked baseline cognitive assessments, particularly when the initial cognitive evaluation took place after MECT had already begun.

### 2.2 Measurements

#### 2.2.1 Primary cognitive ability test (3rd edition, PCAT III)

Cognitive function was assessed using the computerized test tool “Primary Cognitive Ability Test (3rd Edition, PCAT III)”, which underwent standardization across 27 cities and counties in 20 provinces nationwide, covering individuals aged 6 to 90 years. The test has been appraised by the Psychological Measurement Committee of the Chinese Psychological Society and has been found to have appropriate difficulty, and it demonstrates good reliability, validity, discriminant validity, and internal consistency (χ2 = 97.99, df = 25, RMSEA = 0.04), meeting psychometric requirements (25). In this study, cognitive performance was assessed using five adapted tasks corresponding to the key domains of the PCAT: Symbol Search (processing speed), Symmetry Span (working memory), Portrait Memory (episodic memory), Paper Folding (visual-spatial memory), and Vocabulary Tests (language comprehension) (26). These tasks incorporate both established and newly developed paradigms. Specifically, the Symbol Search task and Language comprehension are derived from the Wechsler Intelligence Scales (27), while the Symmetry Span task is adapted from protocols developed by the Engle Laboratory at Georgia Institute of Technology (28). The Portrait Memory task draws on materials from the Clinical Memory Scale (29), and the Paper Folding task is based on the classic construct introduced by Ekstrom et al. (30). The internal consistency, reliability, and structural validity of the five cognitive tests in PCAT III were presented in **Tables 1**, **2**.

**Table 1:** The internal consistency reliability of the five cognitive tests in PCAT III.

Cognitive domain	Test	Adapted source	Cronbach’s α
Processing Speed	Symbol Search	The same task in Wechsler Intelligence Scale	0.96
Working Memory	Symmetry Span	The same task in Engle’s laboratory	—
Episodic Memory	Portrait Memory	The same subtest in the Clinical Memory Scale	0.83
Visual spatial	Paper Folding	The same subtest in the Comprehensive Intelligence Test	0.85
Language Comprehension	Vocabulary Test	The corresponding task in the Wechsler Intelligence Test	0.93

**Table 2:** The structural validity of the five cognitive tests in PCAT III.

Test	Symbol search	Symmetry span	Portrait memory	Paper folding	Vocabulary test
Symbol Search	——				
Symmetry Span	0.43***	——			
Portrait Memory	0.34***	0.50***	——		
Paper Folding	0***	0.45***	0.37***	——	
Vocabulary Test	0.32***	0.41***	0.39***	0.35***	——

***p<0.001.

#### 2.2.2 Hamilton depression rating scale-17 items

HDRS-17 used a 5-point scoring system ranging from 0 to 4, with total scores ranging from 0 to 68 (31). The Chinese version of HDRS-17 was validated and widely used in the measurement of the severity of depressive symptoms and consists of five factors: (1) Anxiety/Somatization/Weight-Loss Factor (items 10, 11, 12, 13, 15, 16); (2) Agitation/Insight Factor (items 9, 17); (3) Depressed Mood/Suicide/Genital Symptoms Factor (items 1, 3, 14); (4) Guilt/Psychomotor Retardation Factor (items 2, 7, 8); and (5) Sleep-Disturbance Factor (items 4, 5, 6) (32).

#### 2.2.3 Hamilton anxiety scale

The severity of anxiety symptoms was assessed using the Hamilton Anxiety Scale (33), a tool that rates each item from 0 to 4, with a total score ranging from 0 to 56. The Chinese version of HAMA consists of seven factors: (1) Anxiety Experiences Factor (items 1, 2, 3); (2) Depressive Symptoms Factor (items 4, 5, 6); (3) Psychosomatic Symptoms Factor (items 7,8); (4) Organ Symptoms Factor (items 9, 10, 11); (5) Genito-urinary Factor (item 12); (6) Autonomic Factor (item 13); (7) Behavior at Interview (item 14) (34).

We also gathered information on the diagnosis, age of onset, prescriptions, and suicide risk from electronic medical records.

### 2.3 Statistical analysis

All data analyses were performed using R 4.4.2. Firstly, we employed Gaussian Mixture Models (GMM) to identify distinct HDRS-17 score trajectories, a semi-parametric approach combining latent class analysis with longitudinal growth modeling (35). Starting with a one-class reference model, we compared two- and three-class solutions with quadratic terms to capture non-linear score patterns. Akaike Information Criterion (AIC), Bayesian Information Criterion (BIC), substantive (entropy), and empirical criteria (enough participants (>5%) occupied each class) were applied to evaluate the models with different clusters. Baseline characteristics were summarized using mean and standard deviation (SD) for continuous variables and counts (percentages) for categorical variables, including demographics, diagnosis, treatment history (MECT/medications), and clinical assessments. Between-group comparisons employed ANOVA or χ² tests, with *post-hoc* analyses for significant differences (p<0.05). Secondly, we assessed depression severity (HDRS-17) and five cognitive abilities using linear mixed-effects model (LMM) with random intercepts. Missing cognitive data were handled via multiple imputation (36) (5 datasets per domain). Models adjusted for demographics and trajectory groups by default, while other covariates were selected through forward selection (p<0.05). Identical modeling procedures were applied to all six outcomes. Fisher’s exact test was used to assess associations between depression trajectory groups and cognitive score distributions across the five cognitive domains.

## 3 Results

### 3.1 Clinical cohort

The medical database included data from 7,868 patients admitted between June 2022 and May 2024. Among these, 792 patients were eligible for data-driven clustering analysis, and 512 patients with complete cognitive testing data were included in the linear mixed-effects model. The number of eligible participants is shown in **Figure 1**.

In total, 792 participants (mean [SD] age, 16.79 [2.57] years) were included in the depressive symptom trajectories. Among the study participants, 73.48% were female, and 62.12% had a high school level education or higher. More than 50% of patients had first-episode depression. A substantial (68.06%) proportion of inpatients were diagnosed with unipolar depression. Descriptive statistics of the study participants are described in **Table 3**.

**Table 3:** Descriptive characteristics of the sample at baseline.

	Participants, no. (%)				P^a^	Comparisons among clusters
	Whole sample	S-r group	M-r group	M-s group		
	N=792 (100.0)	N = 61(7.7)	N = 121 (15.3)	N = 610 (77.0)		
Demographics						
Age, mean (SD) y	16.79(2.57)	17.15 (2.77)	17.17 (2.77)	16.67 (2.51)	0.078	–
Gender					0.585	–
Female	582(73.5)	47(77.0)	85(70.3)	450 (73.8)		
Male	210(26.5)	14 (23.0)	36(29.7)	160 (26.2)		
Education					0.410	–
Elementary School	7(0.9)	0(0.0)	3(2.5)	4(0.6)		
Middle School	293(37.0)	24 (39.3)	44(36.3)	225(36.9)		
High School and higher	492(62.1)	37(60.7)	74(61.2)	381 (62.5)		
Clinical data						
Diagnosis					0.452	–
Unipolar Depression	539(68.1)	44(72.1)	78 (64.5)	417 (68.4)		
Bipolar Depression	79(9.9)	8(13.1)	15(12.4)	56(9.2)		
UBED-CA	174(22.0)	9(14.8)	28(23.1)	137 (22.4)		
Duration, mean (SD) y	3.03(2.24)	3.43 (2.50)	2.90 (2.53)	3.02 (2.14)	0.312	–
Episode Pattern					0.781	–
Single Episode,	398(50.3)	29 (47.6)	59(48.8)	310(50.8)		
Recurrent Episodes	267(33.7)	24(39.3)	44(36.3)	199 (32.6)		
Unknown	127(16.0)	8 (13.1)	18(14.9)	101 (16.6)		
Age of onset, mean (SD) y	13.70(2.68)	13.69 (2.57)	14.27 (2.93)	13.59 (2.63)	0.036	1<(2>3)
Hospitalization times, mean (SD)	1.45(0.81)	1.36 (0.63)	1.43 (0.76)	1.46 (0.84)	0.621	–
Assessment at baseline						
HDRS-17 ^b^, mean (SD)	17.64(6.69)	27.98 (6.35)	16.09 (6.26)	16.91 (5.89)	<0.001	1>(2 = 3)
Factor 1	4.51(3.05)	8.70 (3.13)	3.84 (2.85)	4.22 (2.75)	<0.001	1>(2 = 3)
Factor 2	2.16(1.47)	2.80 (1.57)	1.90 (1.48)	2.14 (1.44)	<0.001	1>(2 = 3)
Factor 3	4.08(1.70)	5.67 (1.77)	3.92 (1.59)	3.95 (1.64)	<0.001	1>(2 = 3)
Factor 4	3.68(1.92)	5.67 (1.80)	3.51 (1.85)	3.51 (1.83)	<0.001	1>(2 = 3)
Factor 5	3.22(2.35)	5.13 (2.18)	2.92 (2.57)	3.09 (2.23)	<0.001	1>(2 = 3)
HAMA ^c^, mean (SD)	13.97(7.94)	22.48 (8.97)	12.42 (7.56)	13.43 (7.40)	<0.001	1>(2 = 3)
Factor 1	4.36(2.70)	6.46 (2.60)	4.21 (2.80)	4.17 (2.60)	<0.001	1>(2 = 3)
Factor 2	5.02(2.48)	7.30 (2.23)	4.55 (2.62)	4.88 (2.36)	<0.001	1>(2 = 3)
Factor 3	0.96(1.46)	1.92 (1.88)	0.69 (1.31)	0.92 (1.40)	<0.001	1>(2 = 3)
Factor 4	1.38(2.03)	2.93 (2.66)	0.99 (1.97)	1.30 (1.90)	<0.001	1>(2 = 3)
Factor 5	0.16(0.52)	0.43 (0.81)	0.07 (0.29)	0.15 (0.51)	<0.001	1>(2 = 3)
Factor 6	0.67(0.96)	1.23 (1.19)	0.54 (0.93)	0.64 (0.92)	<0.001	1>(2 = 3)
Factor 7	1.25(1.13)	1.85 (1.26)	1.25 (1.23)	1.19 (1.08)	<0.001	1>(2 = 3)
Suicidal behavior						
Current Suicide Attempt	107(13.5)	12 (19.7)	17 (14.0)	78 (12.8)	0.319	–
Past Suicide Attempt	27(3.4)	3 (4.9)	5 (4.1)	19(3.1)	0.560	–
Suicidal Ideation Only	368(46.5)	30 (49.2)	52(43.0)	286(46.9)	0.665	–
NSSI	120(15.2)	7.00 (11.5)	15(12.4)	98(16.0)	0.416	–
Treatment data						
Use of MECT	371(46.8)	44 (72.1)	46 (38.0)	281 (46.0)	<0.001	1>(2 = 3)
MECT times, mean (SD)	3.69(4.33)	6.13 (4.76)	2.75 (3.67)	3.63 (4.32)	<0.001	1<(2 = 3)
Use of antidepressants	453(57.2)	45(73.8)	58(47.9)	350(57.4)	0.004	1<(2>3)
Use of stabilizers	187(23.6)	14(23.0)	32(26.5)	141(23.1)	0.714	–
Use of antipsychotics	603(76.1)	49(80.3)	89(73.6)	465(76.2)	0.615	–
Use of BZDs	630(79.6)	58(95.1)	87(71.9)	485(79.5)	<0.001	1>(2 = 3)
Weekly medication Types, mean (SD)	2.36(1.00)	2.72 (0.76)	2.20 (1.03)	2.36 (1.01)	0.004	1>(2 = 3)
Maximum meditation per week, mean (SD)	2.63(1.04)	3.07 (0.70)	2.42 (1.10)	2.63 (1.05)	<0.001	1>(2 = 3)

a One-way ANOVA; Pearson’s Chi-squared test; Fisher’s exact test.

b HDRS-17 consists of five factors. Factor 1: Anxiety/Somatization/Weight-Loss Factor; Factor 2: Agitation/Insight Factor; Factor 3: Depressed Mood/Suicide/Genital Symptoms Factor; Factor 4: Guilt/Psychomotor Retardation Factor; Factor 5: Sleep-Disturbance Factor.

c HAMA consists of seven factors. Factor 1: Anxiety Experiences Factor; Factor 2: Depressive Symptoms Factor; Factor 3: Psychosomatic Factor; Factor 4: Organ Symptoms Factor; Factor 5: Genito-urinary Factor; Factor 6: Autonomic System Factor; Factor 7: Behavior at Interview.

S-R, Severe-Rapid Remission; M-R, Moderate-Rapid Remission; M-S, Moderate-Slow Remission; UBED-CA, Unspecified behavioral and emotional disorders with onset usually occurring in childhood and adolescence; BZDs, Benzodiazepines; NSSI, Non-Suicidal Self-Injury.

**Figure 3:**
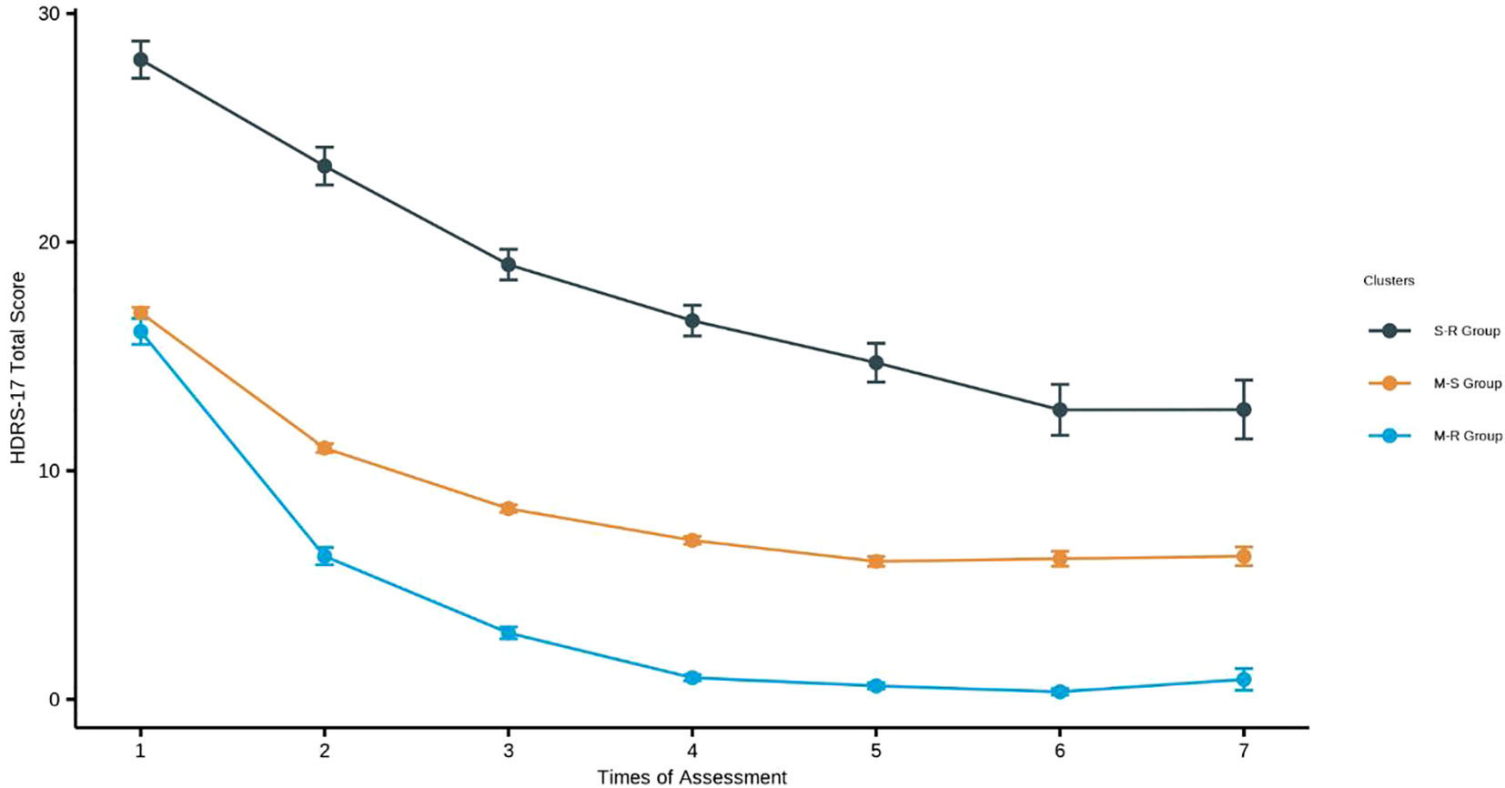
Trajectories of HDRS-17 over 7 weeks using the Gaussian mixture model. M-R Group (Moderate-Rapid Remission Group): Depressive episodes in inpatients with moderate severity symptoms at the beginning and the symptoms remit rapidly. M-S Group (Moderate-Slow Remission Group): Depressive episodes in inpatients with moderate severity symptoms at the beginning and the symptoms remit slowly S-R Group (Severe-Rapid Remission Group): Depressive episodes in inpatients with severe symptoms at the beginning and the symptoms remit rapidly.

**Figure 3** shows the HDRS-17 score over 7 weeks of hospitalization for the three identified groups: Severe-Rapid Remission Group (S-R Group), Moderate-Rapid Remission Group (M-R Group), and Moderate-Slow Remission Group (M-S Group). Each cluster exhibited distinct patterns of symptom change over time. The model fit indices were AIC 23278.70, BIC 23348.82, and entropy 0.66.

The S-R group (n=61, 7.70%, HDRS-17 = 27.98, SD = 6.35) showed significantly greater baseline severity than other groups (all p-values<0.001). Treatment intensity was highest in this group: 72.1% received MECT (mean 6.13 sessions), 73.8% antidepressants, and 95.1% BZDs (all p-values<0.01). The M-R (n=121, 15.28%; HDRS-17 = 16.09, SD = 6.26) and M-S groups (n=610, 77.02%, HDRS-17 = 16.91, SD = 5.89) showed comparable baseline characteristics (all p-values > 0.05). Additional baseline characteristics are presented in **Table 1**.

### 3.2 Cognitive function of the participants within three groups

**Figure 4:**
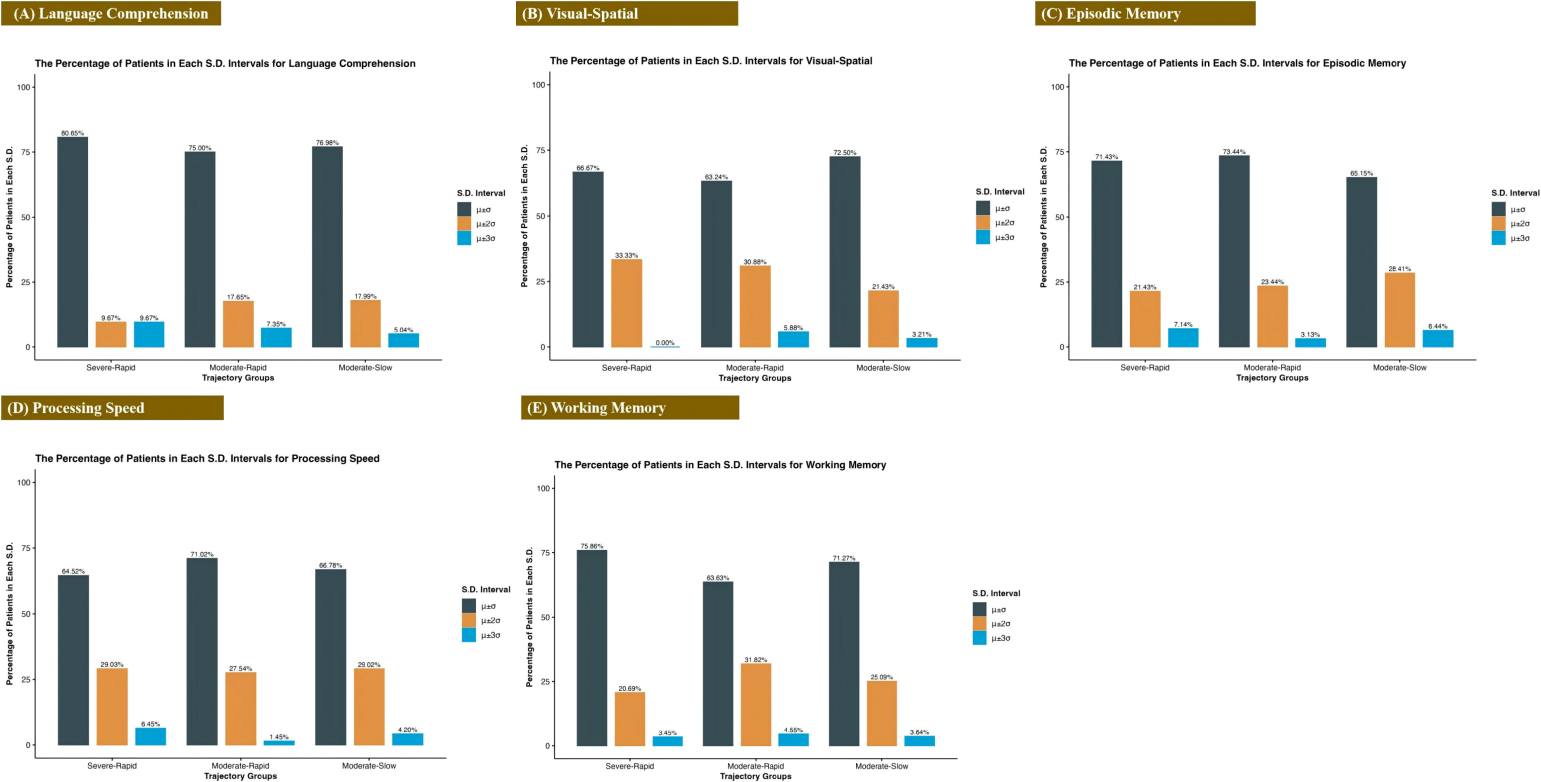
Distribution of cognitive performance across diagnostic groups. Percentage of participants within each group (Severe-Rapid Remission, Moderate-Rapid Remission, Moderate-Slow Remission) scoring in specified standard deviation ranges for five cognitive domains: **(A)** Language Comprehension, **(B)** Visual-Spatial Ability, **(C)** Episodic Memory, **(D)** Processing Speed, and **(E)** Working Memory.

**Figure 4** reveals distinct cognitive performance profiles among the three trajectory groups. In language comprehension, 80.65% of the S-R Group fell within ±1σ, compared to 75.00% in the M-R Group and 76.98% in the M-S Group. The visual-spatial domain showed the most concentrated distribution in the S-R Group, with 100% of scores lying within ±2σ. For working memory, the M-R Group exhibited the lowest proportion of individuals within ±1σ, but the highest within both ±2σ and ±3σ ranges. Similar distributional differences were observed in episodic memory and processing speed. Fisher’s exact tests detected no significant associations between depression trajectory groups and cognitive score distributions across any of the five domains (language comprehension: p = 0.570; visual-spatial: p = 0.349; episodic memory: p = 0.682; working memory: p = 0.681; processing speed: p = 0.752).

### 3.3 Sensitivity analyses for cognitive function by patients with UBED-CA

Analysis of baseline cognitive function in all patients (**Table 4**) and 174 patients with UBED-CA (**Table 5**) revealed nonsignificant trends (all P > 0.05). The raw scores from each test were used in the analysis, with lower values indicating better cognitive performance. The S-R group showed better performance in four domains (visual-spatial ability, episodic memory, working memory, and processing speed), while the moderate-slow remission group had better language comprehension performance compared with other groups.

**Table 4:** Cognitive functions of the 3 clusters for the whole sample at baseline.

Variables	Whole sample	S-r group	M-r group	M-s group	P^a^
	N=510	N=42	N=84	N=384	
Language comprehension, mean (SD)	47.04(12.69)	45.90 (14.13)	47.04 (13.62)	47.17 (12.33)	0.871
Visual-spatial, mean (SD)	6.52(3.57)	5.37 (3.68)	6.54 (3.80)	6.64 (3.49)	0.177
Episodic Memory, mean (SD)	20.65(9.58)	19.21 (10.25)	20.56 (9.22)	20.83 (9.62)	0.696
Working Memory, mean (SD)	3.03(2.55)	2.90 (2.50)	3.17 (2.73)	3.01 (2.52)	0.870
Processing speed, mean (SD)	33.74 (10)	31.90 (10.67)	33.58 (9.32)	33.97 (10.10)	0.545

S-R, Severe-Rapid Remission; M-R, Moderate-Rapid Remission; M-S, Moderate-Slow Remission.

One-way ANOVA; Pearson’s Chi-squared test; Fisher’s exact test.

**Table 5:** Comparison of cognitive functions in the 3 clusters for patients with UBED-CA at baseline.

Variables	Whole sample	S-r group	M-r group	M-s group	P^a^
	N=174	N=9	N=28	N=137	
Language comprehension, mean (SD)	44.06 (13.28)	44.33 (13.37)	47.57 (9.02)	43.33 (13.95)	0.306
Visual-spatial, mean (SD)	5.81 (3.07)	5.11 (3.72)	5.93 (2.92)	5.83 (3.07)	0.775
Episodic Memory, mean (SD)	20.70 (9.58)	20.22 (9.43)	20.79 (8.81)	20.72 (9.81)	0.988
Working Memory, mean (SD)	2.79 (2.63)	1.11 (1.17)	2.79 (2.35)	2.90 (2.73)	0.144
Processing speed, mean (SD)	33.74 (10.20)	32.56 (9.28)	33.50 (7.26)	33.86 (10.80)	0.926

UBED-CA, Unspecified behavioral and emotional disorders with onset usually occurring in childhood and adolescence; S-R, Severe-Rapid Remission; M-R, Moderate-Rapid Remission; M-S, Moderate-Slow Remission.

One-way ANOVA; Pearson’s Chi-squared test; Fisher’s exact test.

### 3.4 Factors associated with depression and cognitive function

**Figure 5:**
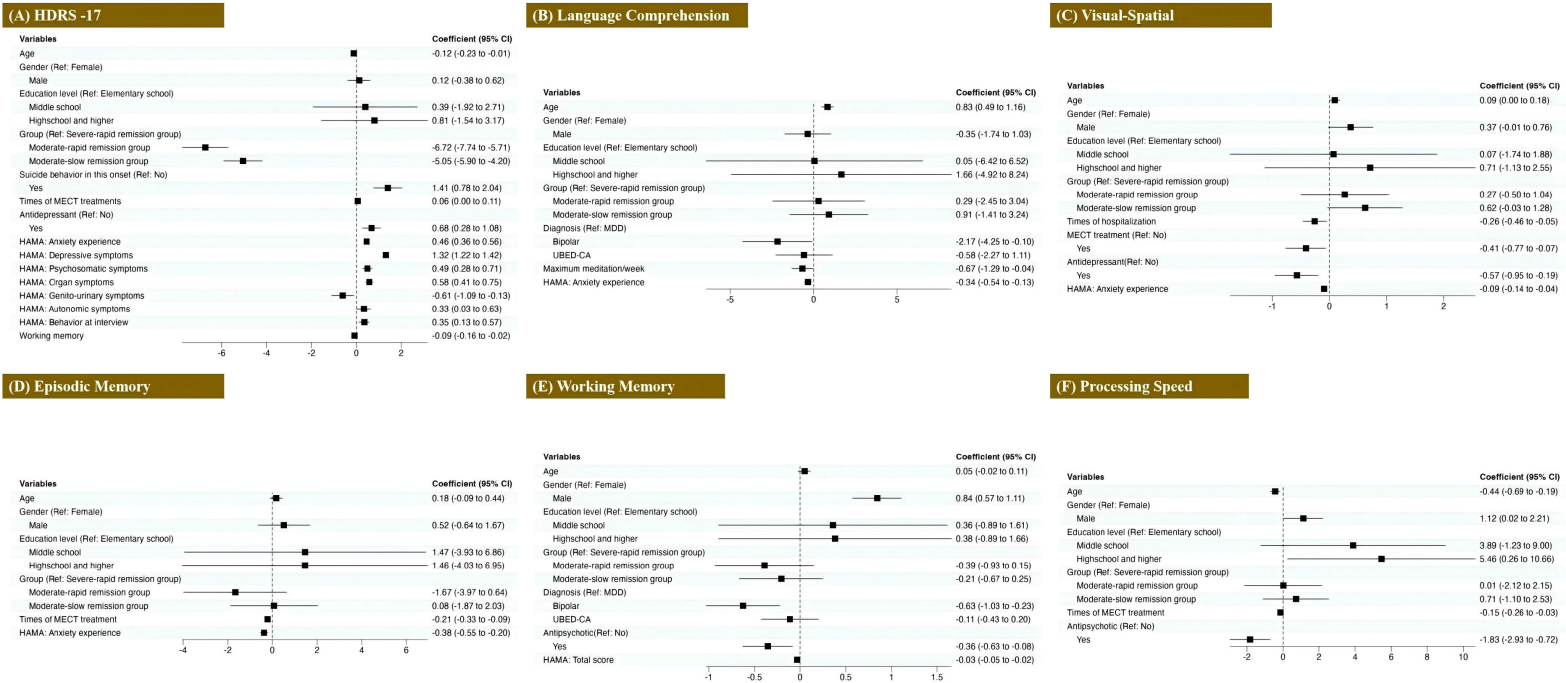
Associations between clinical features and cognitive outcomes in depressive episodes. Forest plots display odds ratios (with 95% confidence intervals) for: **(A)** Depression severity (HDRS-17 scores), **(B)** Visual-Spatial ability, **(C)** Episodic Memory, **(D)** Processing Speed, **(E)** Language Comprehension, and **(F)** Working Memory.

We developed an LMM with the HDRS-17 score as the dependent variable and the raw score of the cognitive test as the independent variable while controlling for covariates such as age, gender, and education level. To further explore the relationship, we also constructed five additional LMMs, reversing the roles of the variables by using the raw scores of different cognitive function assessments as the dependent variables and the HDRS-17 score as the independent variable while maintaining the same covariates. **Figure 5** shows the regression results comparing demographics and clinical features of HDRS-17 and cognitive domains (details in **Supplementary eTable 1-6**).

The severity of depressive symptoms was significantly associated with groups, current suicide attempts, and antidepressant treatment (all p-values < 0.001). It showed positive correlations with all anxiety factor scores except genito-urinary symptoms and the times of MECT (all p-values < 0.05) while exhibiting negative correlations with genito-urinary symptoms and working memory (all p*-*values < 0.05). In terms of cognitive function, older age was associated with better visual-spatial ability (p = 0.041), slower processing speed (*p* < 0.001), and stronger language comprehension (*p* < 0.001). Male participants demonstrated superior processing speed (*p* = 0.045) and working memory (p < 0.001) compared to females, and higher education levels were associated with increased processing speed. Spatial memory exhibited positive correlations with both MECT and antidepressant treatment (all p-values < 0.05), whereas it was negatively associated with hospitalization times and anxiety experience symptoms (all p-values < 0.05). Episodic memory showed significant negative correlations with MECT times and the anxiety experience score (all p-values < 0.001). Processing speed was negatively related to MECT times and antipsychotic treatment (all p-values < 0.001). Language comprehension displayed negative associations with the maximum medication types and anxiety experience (all p-values < 0.05). Compared to patients with unipolar depression, those with bipolar depression exhibited poorer language comprehension (*p* = 0.040) and diminished working memory (*p* = 0.002). Additionally, antipsychotic treatment was negatively associated with working memory (*p* = 0.011) and HAMA scores (*p* < 0.001).

## 4 Discussion

Adolescence is a critical period with a heightened vulnerability to depressive episodes (37), representing a significant global burden as mental health disorders among adolescents and young adults (4, 7, 38). Prior studies have neither examined depression remission trajectories in adolescents nor focused on the critical developmental transition from adolescence to early adulthood. This retrospective cohort study is the first to investigate symptom remission trajectories and associated factors in hospitalized adolescents and young adults with depressive states.

While our findings demonstrate the overall efficacy of intensive treatment protocols, they also reveal important nuances in therapeutic response patterns. Although symptom improvement was achieved across all patient groups (HDRS-17 reduction ≥50%), complete remission remained elusive for a significant subset (23.4%). Notably, the M-R Group demonstrated faster symptom resolution despite receiving less intensive pharmacological intervention (Antidepressants: 47.9% vs. 57.4% in M-S group, p=0.004), suggesting that rapid responders to moderate depressive episodes may benefit more from targeted non-pharmacological approaches such as cognitive behavioral therapy (39) or neuromodulation (40). These findings reveal significant treatment response variability in depressed youth, influenced by unmeasured clinical/environmental factors, particularly psychotherapy access, ward environment, patient characteristics (41), and family involvement (42). These trajectories offer practical long-term guidance by informing key clinical priorities including prognostic stratification, treatment intensity decisions, and optimal intervention timing. Early identification of a patient’s trajectory may help clinicians tailor prognostic communication and personalize treatment plans. For patients on an M-S trajectory, our findings indicate a need for more intensive multimodal therapy with early treatment augmentation rather than a watch-and-wait strategy to achieve better long-term outcomes. Future longitudinal studies should prioritize investigating the dynamic interplay between psychosocial factors, neurodevelopmental trajectories, and treatment engagement to better predict remission patterns.

This study found that the average hospitalization age of patients was 16.79 years, which is the same as the high school age in China. Adolescents exhibit heightened susceptibility to mood and anxiety disorders during this critical developmental period of physiological and psychological transition (43–45). This vulnerability stems from imbalanced maturation of prefrontal-limbic circuits, which creates a temporal gap between heightened emotional reactivity and immature cognitive control (46). Concurrently, exposure to substantial academic stressors further exacerbates their risk for mood disorders (47). Focusing on depressive episodes, Chinese patients demonstrate an earlier mean onset age of 18.8 years (48). The mean onset age of 13.7 years in these hospitalized adolescents and young adults was significantly earlier than reported in prior studies. While partly reflecting our recruitment criteria, this earlier onset also suggests greater disease severity and functional impairment in youth requiring intensive treatment. Moreover, Earlier onset was associated with slower improvement in depressive symptoms (*p* < 0.05). This suggests that the age of onset may be a significant factor for remission trajectory.

In all three groups, the proportion of females exceeded that of males (77% vs. 23%, 70.3% vs. 29.7%, 73.8% vs. 26.2%, *p* = 0.585), consistent with established gender patterns in mood disorders (49). Gender differences in depressive symptoms peak at 16–19 years, slightly later than in Major depressive disorder (50, 51), consistent with our findings. These results indicate that gender disparities in depressive symptoms first appear in adolescence, peak in early adulthood, and manifest more prominently in females. Asian female adolescents and young adults showed elevated depression risk (5), shaped by biological (52) and economic (50) factors. These findings underscore a critical developmental period where neurobiological vulnerability and environmental stressors jointly increase susceptibility, particularly in this population.

Suicide-related behaviors showed comparable rates across all three groups during current and past episodes, with current depression severity significantly linked to suicidal risk (OR = 1.41, 95% CI 0.78-2.04). It is similar to the previous study (53) and underscores the urgency of intervention, given the leading cause of death in 15–24 year olds (54). While youth suicide prevention research faces methodological and ethical barriers, our data reveal that intensive treatment models effectively address this crisis: the S-R group’s marked symptom reduction (72.1% receiving MECT plus with higher antidepressant and BZD prescription rates, all p-values<0.001) demonstrates how acute-phase strategies can mitigate risk through rapid symptom control, mirroring adult protocols (55). This supports implementing targeted screening with stepped-care referrals to intensive therapies for high-risk youth.

Previous research has shown that patients in major depressive episodes reliably show impaired cognition about learning, executive control, processing speed, memory, and attention (56–58). Multiple studies confirm that BD patients exhibit deficits across several cognitive domains, particularly verbal memory, attention, and executive function (59–61). Furthermore, this dysfunction in BD appears to show dose-dependent worsening with episode recurrence (62). While all groups exhibited similar mean cognitive scores, the S-R Group demonstrated significantly greater variability (± 2-3σ) in processing speed and visual-spatial abilities, which represent cognitive domains particularly vulnerable to depression severity. In contrast, language comprehension showed the most consistent performance across groups (predominantly within ±1σ), indicating this domain may be more resistant to acute symptom fluctuations. Our results suggest that trajectories of depression symptoms appeared to be independent of baseline cognitive function (all p > 0.05). This may indicate that, within the acute treatment phase (6–8 weeks), the rate of symptomatic improvement could be more closely related to disease-specific characteristics and treatment modalities than to cognitive performance. However, it should be noted that acute factors such as motivational variability, fatigue, and depressive state, particularly relevant in hospitalized adolescents, may have introduced noise or confounding effects in cognitive assessment.

After adjusting for age, sex, education level, and group assignment in a multiple regression model, working memory demonstrated a statistical association with depression severity, whereas non-significant associations observed in other cognitive domains should be interpreted with caution. These findings may reflect a true absence of effects, but could also stem from limited statistical power in the current study, particularly given the moderate sample size and multiple comparisons conducted. Additionally, differences in the proportion of 16-year-old participants across groups may have influenced working memory assessments. Executive function development peaks around age 16 (63), making high-difficulty working memory tasks particularly sensitive within this age group. Future studies with larger and more balanced samples are warranted to clarify the relative contributions of various cognitive domains to depression severity and to validate the specificity of the working memory association.

The observed dissociation between processing speed decline (OR = -0.44) and language comprehension improvement (OR = 0.83) with aging demonstrates domain-specific cognitive dysfunction that varies by development stage. In adolescents and young adults, processing speed declined with advancing age, whereas language comprehension showed age-related improvement. Notably, all cognitive domains correlated with baseline anxiety levels, contrasting with traditional models where anxiety typically precedes cognitive decline by approximately two years in elderly populations (64). It suggests that anxiety symptoms may exert greater influence on cognitive performance than depressive symptoms during early-life mood episodes. While cognitive aging research has predominantly examined adults and elderly individuals (65–69), our retrospective analytical approach provides novel methodological insights for studying neurodevelopmental trajectories in younger populations. Regarding cognitive differences between unipolar and bipolar depression, our preliminary analysis suggests that working memory and language comprehension may be particularly associated with bipolar depression. It is plausible that early-stage bipolar depression preferentially affects these cognitive domains. Further studies with larger samples are needed to validate and generalize these findings.

The study has several limitations. First, no significant predictors differentiated M-R Group from M-S Group despite comparable baseline symptom severity, treatment, and demographic/clinical characteristics (all p-values > 0.05). This unexplained variance may implicate unmeasured factors [i.e., genetic susceptibility (70, 71)] and early life stress (71–73), which warrants investigation in future studies. Second, the short study duration and absence of post-discharge follow-up prevented assessment of functional outcomes beyond the acute phase. Patients with adolescent and early-adulthood-onset depression exhibit stronger associations with adult social dysfunction (74), greater social burden (75), and prolonged recovery periods. Early in 2014, researchers called for greater attention to quality-of-life outcomes in depression management, marking a paradigm shift toward functional recovery as a key treatment goal. Within this framework, preserved social functioning emerges as a fundamental component of life quality enhancement, particularly for adolescent-onset cases. Addressing these needs demands coordinated efforts across scientific, clinical, and societal domains. Third, cognitive function was assessed solely at baseline, limiting insight into its progression or association with remission trajectories. Although these tools are widely used in research across age groups, some were originally normed on adult populations. This might affect the precision of comparing their performance to adolescent-specific norms. Future studies should implement longitudinal designs to track dynamic associations between cognitive performance, depressive symptom improvement, and academic functional recovery, while incorporating additional measures to capture a broader spectrum of cognitive dysfunction in this population.

## 5 Conclusion

This study identified three remission trajectories among adolescents receiving intensive inpatient treatment, suggesting that structured protocols may hold clinical value. Working memory was associated with depression severity, while anxiety correlated more strongly with general cognitive impairment than depression. Bipolar depression was linked to poorer language and working memory, though these potentially subtype-relevant findings remain preliminary and require further validation. The results justify further research into individualized approaches emphasizing anxiety and cognitive support. However, these insights stem from retrospective inpatient data and require validation in prospective studies. Larger and more diverse cohorts are needed to verify the generalizability and diagnostic relevance of these cognitive patterns.

## Data Availability

The original contributions presented in the study are included in the article/**Supplementary Material**. Further inquiries can be directed to the corresponding author.
