# Supplementary material for: Remission trajectories and cognitive function in hospitalized youth with depressive episodes
Source: Front Psychiatry. 2025 Oct 1;16:1673240. doi: 10.3389/fpsyt.2025.1673240 (PMC12521248; doi:10.3389/fpsyt.2025.1673240)
Supplement: Supplementary file 1 [file Table1.docx]

**eTable 1** LMM for the Severity of Depression in Patients with Depressive Episodes

|  | **Coefficient** | **S.E.** | **t** | **d.f.** | ***p*-value** | **95% CI** |
| --- | --- | --- | --- | --- | --- | --- |
| Intercept | 10.199 | 1.508 | 6.76 | 730.4 | <.001 | (7.238, 13.160) |
| Age | -0.12 | 0.058 | -2.06 | 705.8 | 0.039 | (-0.234, -0.006) |
| Gender (Ref: Female) |  |  |  |  |  |  |
| Male | 0.122 | 0.255 | 0.48 | 721.1 | 0.631 | (-0.378, 0.622) |
| Education level (Ref: Elementary school) |  |  |  |  |  |  |
| Middle school | 0.393 | 1.18 | 0.33 | 700.3 | 0.739 | (-1.923, 2.710) |
| High school and higher | 0.815 | 1.20 | 0.68 | 703.4 | 0.498 | (-1.542, 3.171) |
| Group (Ref: Severe-rapid remission group) |  |  |  |  |  |  |
| Mild-rapid remission group | -6.724 | 0.516 | -13.03 | 799.5 | <.001 | (-7.737, -5.712) |
| Mild-slow remission group | -5.051 | 0.433 | -11.66 | 766.4 | <.001 | (-5.901, -4.200) |
| Current Suicide Attempt (Ref: No) |  |  |  |  |  |  |
| Yes | 1.41 | 0.320 | 4.4 | 750.4 | <.001 | (0.781, 2.039) |
| Times of MECT | 0.055 | 0.026 | 2.08 | 696.7 | 0.038 | (0.003, 0.107) |
| Antidepressant (Ref: No) |  |  |  |  |  |  |
| Yes | 0.679 | 0.206 | 3.3 | 1276 | <.001 | (0.276, 1.082) |
| HAMA: Anxiety experience | 0.459 | 0.053 | 8.7 | 2684.1 | <.001 | (0.356, 0.563) |
| HAMA: Depression symptom | 1.321 | 0.052 | 25.57 | 2835.5 | <.001 | (1.220, 1.422) |
| HAMA: Physiosomatic symptoms | 0.494 | 0.108 | 4.57 | 2858.6 | <.001 | (0.282, 0.706) |
| HAMA: Organ symptoms | 0.579 | 0.086 | 6.76 | 2946 | <.001 | (0.411, 0.747) |
| HAMA: Genito-urinary symptoms | -0.611 | 0.246 | -2.48 | 2792.3 | 0.013 | (-1.094, -0.128) |
| HAMA: Autonomic symptoms | 0.331 | 0.154 | 2.14 | 2835.7 | 0.032 | (0.028, 0.634) |
| HAMA: behavior at interview | 0.351 | 0.114 | 3.07 | 2673.4 | 0.002 | (0.126, 0.575) |
| Working memory | -0.088 | 0.035 | -2.52 | 3040.5 | 0.012 | (-0.156, -0.019) |

**eTable 2** LMM for Language Apprehension in Patients with Depressive Episodes

|  | **Coefficient** | **S.E.** | **t** | **d.f.** | ***p*-value** | **95% CI** |
| --- | --- | --- | --- | --- | --- | --- |
| Intercept | 34.04 | 4.40 | c | 799.9 | <.001 | (25.399, 42.682) |
| Age | 0.83 | 0.17 | 4.85 | 785.6 | <.001 | (0.493, 1.164) |
| Gender (Ref: Female) |  |  |  |  |  |  |
| Male | -0.35 | 0.70 | -0.50 | 787.2 | 0.614 | (-1.735, 1.026) |
| Education level  (Ref: Elementary school) |  |  |  |  |  |  |
| Middle school | 0.05 | 3.30 | 0.02 | 783.4 | 0.988 | (-6.421, 6.523) |
| High school and higher | 1.66 | 3.35 | 0.49 | 786.6 | 0.621 | (-4.923, 8.240) |
| Group (Ref: Severe-rapid remission group) |  |  |  |  |  |  |
| Mild-rapid remission group | 0.29 | 1.40 | 0.21 | 832.6 | 0.835 | (-2.453, 3.036) |
| Mild-slow remission group | 0.91 | 1.18 | 0.77 | 818.0 | 0.441 | (-1.410, 3.237) |
| Diagnosis (Ref: MDD) |  |  |  |  |  |  |
| Bipolar | -2.17 | 1.06 | -2.06 | 770.8 | 0.040 | (-4.247, -0.100) |
| UBED-CA | -0.58 | 0.86 | -0.67 | 791.9 | 0.504 | (-2.268, 1.115) |
| Maximum meditation/week | -0.67 | 0.31 | -2.11 | 802.6 | 0.035 | (-1.286, -0.045) |
| HAMA: Anxiety experience | -0.34 | 0.11 | -3.20 | 2979.7 | 0.001 | (-0.541, -0.130) |

**eTable 3** LMM for Visual-spatial in Patients with Depressive Episodes

|  | **Coefficient** | **S.E.** | **t** | **d.f.** | ***p*-value** | **95% CI** |
| --- | --- | --- | --- | --- | --- | --- |
| Intercept | 5.09 | 1.18 | 4.32 | 802.70 | <.001 | (2.779, 7.402) |
| Age | 0.09 | 0.05 | 2.05 | 785.10 | 0.041 | (0.004, 0.184) |
| Gender (Ref: Female) |  |  |  |  |  |  |
| Male | 0.37 | 0.20 | 1.89 | 786.00 | .059 | (-0.015, 0.758) |
| Education level (Ref: Elementary school) |  |  |  |  |  |  |
| Middle school | 0.07 | 0.92 | 0.08 | 782.10 | 0.938 | (-1.738, 1.883) |
| High school and higher | 0.71 | 0.94 | 0.76 | 785.00 | 0.448 | (-1.129, 2.551) |
| Group (Ref: Severe-rapid remission group) |  |  |  |  |  |  |
| Mild-rapid remission group | 0.27 | 0.39 | 0.69 | 828.50 | 0.493 | (-0.500, 1.036) |
| Mild-slow remission group | 0.62 | 0.33 | 1.88 | 814.80 | 0.061 | (-0.028, 1.276) |
| Times of hospitalization | -0.26 | 0.11 | -2.45 | 826.40 | 0.015 | (-0.464, -0.051) |
| MECT treatment (Ref: No) |  |  |  |  |  |  |
| Yes | -0.42 | 0.18 | -2.33 | 787.80 | 0.02 | (-0.765, -0.066) |
| Antidepressant (Ref: No) |  |  |  |  |  |  |
| Yes | -0.57 | 0.19 | -2.97 | 1490.30 | 0.003 | (-0.951, -0.194) |
| HAMA: Anxiety experience | -0.09 | 0.03 | -3.34 | 3028.10 | <.001 | (-0.145, -0.038) |

**eTable 4** LMM for Episodic Memory in Patients with Depressive Episodes

|  | **Coefficient** | **S.E.** | **t** | **d.f.** | ***p*-value** | **95% CI** |
| --- | --- | --- | --- | --- | --- | --- |
| Intercept | 19.25 | 3.49 | 5.52 | 795.5 | <.001 | (12.397, 26.092) |
| Age | 0.18 | 0.14 | 1.32 | 784.5 | 0.188 | (-0.087, 0.444) |
| Gender (Ref: Female) |  |  |  |  |  |  |
| Male | 0.52 | 0.59 | 0.88 | 786.0 | 0.381 | (-0.639, 1.672) |
| Education level (Ref: Elementary school) |  |  |  |  |  |  |
| Middle school | 1.47 | 2.75 | 0.53 | 782.0 | 0.594 | (-3.934, 6.863) |
| High school and higher | 1.46 | 2.80 | 0.52 | 785.0 | 0.601 | (-4.027, 6.953) |
| Group (Ref: Severe-rapid remission group) |  |  |  |  |  |  |
| Mild-rapid remission group | -1.67 | 1.18 | -1.42 | 832.6 | 0.157 | (-3.970, 0.641) |
| Mild-slow remission group | 0.08 | 0.99 | 0.08 | 817.1 | 0.939 | (-1.874, 2.027) |
| Times of MECT | -0.21 | 0.06 | -3.39 | 775.1 | <.001 | (-0.326, -0.087) |
| HAMA: Anxiety experience | -0.38 | 0.09 | -4.33 | 2988.7 | <.001 | (-0.545, -0.205) |

**eTable 5** LMM for Working Memory in Patients with Depressive Episodes

|  | **Coefficient** | **S.E.** | **t** | **d.f.** | ***p*-value** | **95% CI** |
| --- | --- | --- | --- | --- | --- | --- |
| Intercept | 2.68 | 0.85 | 3.15 | 822.1 | 0.002 | (1.009, 4.357) |
| Age | 0.05 | 0.03 | 1.35 | 774.4 | 0.176 | (-0.020, 0.110) |
| Gender (Ref: Elementary school) |  |  |  |  |  |  |
| Male | 0.84 | 0.14 | 6.19 | 777.5 | <.001 | (0.575, 1.110) |
| Education level (Ref: Elementary school) |  |  |  |  |  |  |
| Middle school | 0.36 | 0.64 | 0.56 | 782.6 | 0.574 | (-0.893, 1.611) |
| High school and higher | 0.38 | 0.65 | 0.59 | 784.8 | 0.555 | (-0.890, 1.657) |
| Group (Ref: Severe-rapid remission group) |  |  |  |  |  |  |
| Mild-rapid remission group | -0.39 | 0.28 | -1.42 | 872.2 | 0.155 | (-0.932, 0.149) |
| Mild-slow remission group | -0.21 | 0.23 | -0.89 | 846.4 | 0.372 | (-0.666, 0.249) |
| Diagnosis (Ref: MDD) |  |  |  |  |  |  |
| Bipolar | -0.63 | 0.21 | -3.06 | 766.0 | 0.002 | (-1.028, -0.225) |
| UBED-CA | -0.11 | 0.16 | -0.71 | 780.6 | 0.48 | (-0.426, 0.201) |
| Antipsychotic (Ref: No) |  |  |  |  |  |  |
| Yes | -0.36 | 0.14 | -2.56 | 1334.4 | 0.011 | (-0.629, -0.083) |
| HAMA total score | -0.03 | 0.01 | -4.48 | 2947.4 | <.001 | (-0.046, -0.018) |

**eTable 6** LMM for Processing Speed in Patients with Depressive Episodes

|  | **Coefficient** | **S.E.** | **t** | **d.f.** | ***p*-value** | **95% CI** |
| --- | --- | --- | --- | --- | --- | --- |
| Intercept | 37.81 | 3.31 | 11.43 | 792.4 | <.001 | (31.322, 44.307) |
| Age | -0.44 | 0.13 | -3.44 | 790.1 | <.001 | (-0.692, -0.189) |
| Gender (Ref: Female) |  |  |  |  |  |  |
| Male | 1.12 | 0.56 | 2.00 | 791 | 0.045 | (0.023, 2.212) |
| Education level (Ref: Elementary school) |  |  |  |  |  |  |
| Middle school | 3.89 | 2.61 | 1.49 | 787.8 | 0.136 | (-1.225, 9.004) |
| High school and higher | 5.46 | 2.65 | 2.06 | 790.6 | 0.040 | (0.258, 10.658) |
| Group (Ref: Severe-rapid remission group) |  |  |  |  |  |  |
| Mild-rapid remission group | 0.01 | 1.09 | 0.01 | 789.8 | 0.991 | (-2.124, 2.149) |
| Mild-slow remission group | 0.72 | 0.93 | 0.77 | 786.4 | 0.440 | (-1.102, 2.532) |
| Times of MECT | -0.15 | 0.06 | -2.54 | 787.1 | 0.011 | (-0.262, -0.033) |
| Antipsychotic (Ref: No) |  |  |  |  |  |  |
| Yes | -1.83 | 0.56 | -3.25 | 1374.3 | 0.001 | (-2.929, -0.725) |
